# *NDNF* variants are rare in patients with congenital hypogonadotropic hypogonadism

**DOI:** 10.1038/s41439-021-00137-x

**Published:** 2021-02-02

**Authors:** Satoshi Tamaoka, Erina Suzuki, Atsushi Hattori, Tsutomu Ogata, Maki Fukami, Yuko Katoh-Fukui

**Affiliations:** 1 Department of Molecular Endocrinology, National Research Institute for Child Health and Development, Tokyo, Japan; 2 Department of Pediatrics, Hamamatsu University School of Medicine, Hamamatsu, Japan

**Keywords:** Neuroendocrine diseases, Endocrine reproductive disorders, Rare variants

## Abstract

Although *NDNF* was recently reported as a novel causative gene for congenital hypogonadotropic hypogonadism (CHH), this conclusion has yet to be validated. In this study, we sequenced *NDNF* in 61 Japanese CHH patients. No variants, except for nine synonymous substitutions that appear to have no effect on splice-site recognition, were identified in *NDNF* coding exons or flanking intronic sequences. These results indicate the rarity of *NDNF* variants in CHH patients and highlight the genetic heterogeneity of CHH.

Congenital hypogonadotropic hypogonadism (CHH) is a rare condition that results in hypomasculinization of male neonates and delayed puberty in children of both sexes^1^. CHH occurs as a component of malformation syndromes, such as Kallmann syndrome (MIM: 308700, 614897, and 613301), CHARGE syndrome (MIM: 214800), and septo-optic dysplasia (SOD) (MIM: 182230), though it can also manifest as an isolated endocrine disorder^1^. To date, more than 30 genes have been implicated in CHH; however, pathogenic variants in these genes together account for only approximately half of all cases^1^. This suggests that other genes are also involved in the development of CHH. In 2020, Messina et al. performed whole-exome sequencing for 240 unrelated CHH patients mostly of European origin and identified four apparently pathogenic variants in *NDNF* (NM_024574.4) (p. Lys62*, p. Tyr128Thrfs*55, p. Trp469*, and p. Thr201Ser) in four patients^2^. The four patients presented with Kallmann syndrome and carried no pathogenic variants in other known CHH-causing genes. Moreover, the four *NDNF* variants were shared by family members who exhibited anosmia/hyposmia or delayed puberty. Furthermore, the authors showed that neuron-derived neurotrophic factor, which is encoded by *NDNF/Ndnf*, is involved in the survival, migration, and growth of neurons in mice. These results provide the first indication that *NDNF* is a causative gene in CHH. However, because there are no additional reports of *NDNF* pathogenic variants in humans, the clinical significance of *NDNF* abnormalities remains unclear.

In this study, we performed mutation screening of *NDNF* in 61 Japanese patients clinically diagnosed with CHH. This study was approved by the Institutional Review Board Committee at the National Center for Child Health and Development (#512), and informed consent was obtained from the participants or their parents. The participants exhibited isolated normosmic HH (*n* = 20), combined pituitary hormone deficiency (CPHD, *n* = 20), Kallmann syndrome (*n* = 16), SOD (*n* = 4), or CHARGE syndrome (*n* = 1). Most of these patients were identified by delayed puberty, which is defined as the lack of pubertal signs at 15 years of age in males and at 13 years of age in females^3^, or by hypomasculinized external genitalia at birth. The remaining cases were diagnosed with CHH during endocrinological evaluations for CPHD, SOD, or CHARGE syndrome. Genomic DNA was extracted from peripheral blood samples. Prior to this study, sequence analysis of the nine major causative genes for CHH (*ANOS1*, *CHD7*, *FGFR1*, *FGF8*, *GNRHR*, *GNRH1*, *KISS1R*, *PROKR2*, and *TACR3*) was performed for all except for 13 (eight with CPHD, two with isolated normosmic HH, two with SOD, and one with Kallmann syndrome) cases^4^. The 48 participants were confirmed to carry no pathogenic variants in the nine genes. In the present study, we analyzed all coding exons (exons 2–4) and flanking intronic sequences of *NDNF* by direct sequencing. The primer sequences used are available upon request. Then, we referred to the gnomAD database (https://gnomad.broadinstitute.org/) to examine the allele frequencies of the identified variants in the general population. We also consulted the combined annotation-dependent depletion program (CADD; https://cadd.gs.washington.edu/snv), the Human Splicing Finder program (https://hsf.genomnis.com/home), the Alternative Splice Site Predictor program (http://wangcomputing.com/assp/), the NNSPLICE program (http://fruitfly.org/seq_tools/splice.html), and the SpliceRover program (http://bioit2.irc.ugent.be/splicerover/) to predict the effect of each variant on protein function and splice-site recognition.

Except for nine synonymous substitutions, we detected no variants in the coding exons or exon–intron boundaries of *NDNF* (Table 1). Of the nine variants, seven are common SNPs with allele frequencies in the general population of more than 10%. The remaining two substitutions, NM_024574.4:c.1029C>T (p. Val343=) and c.1035A>G (p. Leu345=), are rare variants with allele frequencies in the gnomAD database of 4.395 × 10^−4^ and 7.103 × 10^−6^, respectively. The nine variants invariably showed low CADD scores and appeared to have negligible effects on splice-site recognition (Table 1). This suggests that all these variants are functionally neutral polymorphisms.

**Table 1:** *NDNF* variants identified in the present study.

Variant			Number of patients with the variant (*n* = 61)		Allele frequency in the general population^b^	Functional assessment^c^	Effect on splicing			
Nucleotide change	Amino acid change	dbSNP^a^	Homozygote	Heterozygote			HSF^d^	ASSP^e^	NNSPLICE^f^	SpliceRover^g^
c.186G>A	p.Lys62=	rs2276959	61	0	0.801	10.690	No effect	No effect	No effect	No effect
c.429C>T	p.Ser143=	rs3733560	16	29	0.520	1.401	Potential alteration	No effect	No effect	No effect
c.435A>G	p.Leu145=	rs3733559	2	28	0.188	1.083	Potential alteration	Cryptic donor (5.80)	No effect	Cryptic donor (0.30)
c.939T>C	p.Asp313=	rs3822230	18	30	0.551	3.090	No effect	No effect	No effect	No effect
c.1029C>T	p.Val343=	rs145661282	0	1	4.395 × 10^−4^	0.016	Potential alteration	No effect	No effect	No effect
c.1035A>G	p.Leu345=	rs3733558	0	1	7.103 × 10^−6^	0.067	No effect	No effect	No effect	No effect
c.1035A>C	p.Leu345=	rs3733558	20	28	0.547	0.057	Potential alteration	No effect	No effect	No effect
c.1392A>G	p.Ser464=	rs1397645	58	3	0.831	0.448	No effect	No effect	No effect	No effect
c.1567C>T	p.Leu523=	rs34766411	2	25	0.252	8.121	Potential alteration	No effect	No effect	No effect

^a^Single Nucleotide Polymorphism Database: https://www.ncbi.nlm.nih.gov/snp/.

^b^Genome Aggregation Database: https://gnomad.broadinstitute.org/.

^c^CADD: https://cadd.gs.washington.edu/.

^d^HSF: Human Splicing Finder: http://www.umd.be/HSF3/.

^e^ASSP: Alternative Splice Site Predictor: http://wangcomputing.com/assp/. Default threshold: 4.50.

^f^NNSPLICE: http://fruitfly.org/seq_tools/splice.html. Default threshold: 0.40.

^g^SpliceRover: http://bioit2.irc.ugent.be/splicerover/. Default threshold: 0.10.

These findings indicate that *NDNF* pathogenic variants are uncommon among Japanese patients with CHH. Our data are consistent with a previous report from Europe^2^. Thus, the contribution of *NDNF* variants to the etiology of CHH appears to be small. Notably, most of our subjects were confirmed to have no pathogenic variants in major known CHH-causing genes; in these cases, CHH may have resulted from unique genetic or epigenetic abnormalities. Indeed, it is possible that several genetic factors involved in CHH remain undetermined.

Altogether, the results of this study, in conjunction with those of the previous study by Messina et al.^2^, indicate that pathogenic *NDNF* variants are rare in CHH patients of both European and Asian descent. These data emphasize the genetic heterogeneity of CHH.

## References

[1] Young J (2019). Clinical management of congenital hypogonadotropic hypogonadism. Endocr. Rev..

[2] Messina A (2020). Neuron-derived neurotrophic factor is mutated in congenital hypogonadotropic hypogonadism. Am. J. Hum. Genet..

[3] The MHLW study group for hypothalamic and pituitary dysfunction. (2019). The diagnosis and treatment guidelines for hypothalamic and pituitary dysfunction (in Japanese). Jpn. J. Endocrinol..

[4] Topaloğlu AK (2017). Update on the genetics of idiopathic hypogonadotropic hypogonadism. J. Clin. Res. Pediatr. Endocrinol..

